# Effects of Lithium Carbonate and Superplasticizer on Ultra-Early Strength of Alite-Ye’elimite-Belite-Ferrite Cement

**DOI:** 10.3390/ma17081742

**Published:** 2024-04-10

**Authors:** Peng Du, Hao Sun, Xiaolei Lu, Yongbo Huang, Xin Cheng

**Affiliations:** 1 Provincial Key Laboratory of Preparation and Measurement of Building Materials, University of Jinan, Jinan 250022, China; mse_dup@ujn.edu.cn (P.D.); mse_luxl@ujn.edu.cn (X.L.); mse_huangyb@ujn.edu.cn (Y.H.); 2 Shandong Luqiao Building Materials Co., Ltd., Jinan 250000, China; 13853172340@163.com

**Keywords:** alite-ye’elimite-belite-ferrite cement, Li_2_CO_3_, superplasticizer, ultra-early strength, micro-analysis

## Abstract

Alite-ye’elimite-belite-ferrite cement (AYBFC) integrates the advantages of calcium sulfoaluminate cement and Portland cement, but its ultra-early strength needs to be further improved when applied to rush repair and construction works. In this study, the ultra-early strength of AYBFC was improved using lithium carbonate (Li_2_CO_3_) and superplasticizer. The results showed that an appropriate amount of Li_2_CO_3_ could significantly improve the ultra-early strength of AYBFC, since it was capable of promoting the hydration reaction of AYBFC. After polycarboxylate superplasticizer was doped on this basis, the ultra-early compressive strength of AYBFC was further improved. This was because the superplasticizer could markedly enhance the matrix compactness despite its inhibitory effect on the hydration reaction of cement and the generation of hydration products.

## 1. Introduction

Some concrete structures need to be urgently repaired because of sudden damage, such as pipeline leakage and traffic road damage, which requires a short time and high timeliness. Calcium sulfoaluminate cement (CSA) has the characteristics of fast hardening, early strength, high corrosion resistance, and high permeability resistance [1], and is considered to be a potential rapid repair engineering material. Some scholars have used additional components to further speed up the hydration rate and improve the ultra-early strength of CSA. Gypsum can improve the early hydration rate of CSA, and at a low dose, it can effectively improve the early compressive strength and reduce the drying shrinkage. By contrast, with a dose that is too high, the later strength of the hardened cement paste will decline [2]. Lithium salts make it possible for CSA to skip over the hydration induction period and directly enter the hydration acceleration period, thus improving the early hydration rate and hydration heat release [3,4,5]. However, an excess dose of lithium salts will affect the later compressive and flexural strength of cement. In addition, some scholars [6,7,8] have explored the effects of mineral admixtures on the early hydration behavior, mechanical properties, and shrinkage performance of CSA.

However, the above studies are only from the perspective of additional components to improve the hydration rate and ultra-early mechanical properties of calcium sulphoaluminate cement, but its shortcomings of low alkalinity, poor bonding properties, and poor development continuity of long-term mechanical properties need to be solved from the perspective of mineral phase composition design. Alite-ye’elimite-belite-ferrite cement (AYBFC) [9,10,11,12], which has come into being in recent years, not only has the characteristics of CSA, but also possesses the advantages of Portland cement, such as high alkalinity, high bonding properties due to the formation of more gel hydration products, the stable development of long-term mechanical properties, etc. Therefore, AYBFC is very suitable for rush repair and construction.

AYBFC can be prepared by various methods. It is an effective method to use C_3_S mineral as the seed to induce more C_3_S to form in CSA; for example, the 7 wt.% C_3_S seed can induce 13 wt.% new C_3_S to form in CSA [10], but it is very difficult to use this method for the actual production of AYBFC. The AYBFC clinker has been successfully calcined by the technology of barium ion doping and liquid phase control [11,12], and the cement has been produced by some companies [13]. When the gypsum dosage is 15 wt.%, the AYBFC reaches the highest compressive strength [12].

However, the effects of additives commonly used in CSA on AYBFC have not been reported yet. This study aims to probe into the effects of Li_2_CO_3_ and superplasticizer on the ultra-early strength of AYBFC to put forward an improvement method for its ultra-early strength, expecting to provide a path for a better application of AYBFC in rush repair and construction works.

## 2. Materials and Methods

### 2.1. Raw Materials

**Figure 1:**
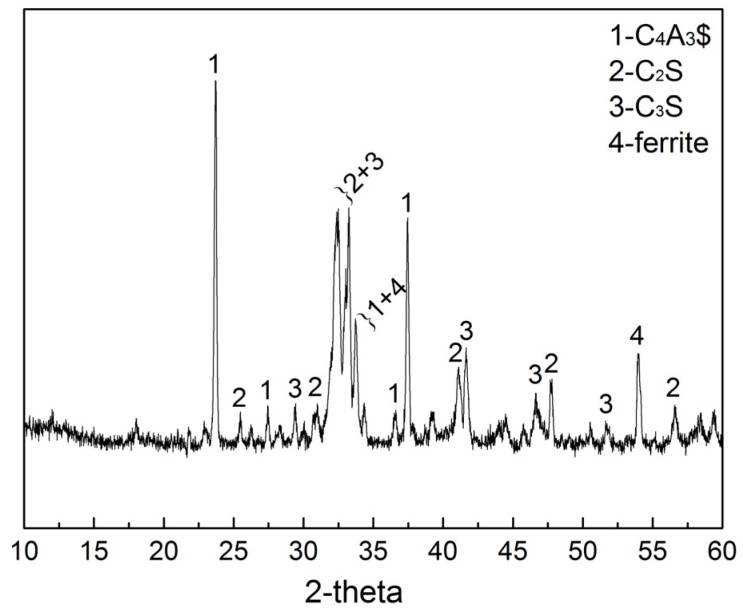
Phase analysis of AYBFC clinker.

In this study, AYBFC produced by Shandong Linqu Shengwei Special Cement Co., Ltd., Weifang, China, was adopted, which was composed of 15 wt.% gypsum and 85 wt.% clinker., with its clinker phase analysis in Figure 1, its clinker mineral composition quantitatively analyzed by using the method of Rietveld refinement shown in Table 1, and its basic properties shown in Reference [12]. The model of Li_2_CO_3_ used in the experiment was MACKLIN L812282, with a purity of 99.5%. The polycarboxylate superplasticizer used was produced by Jiangsu SBT New Materials Co., Ltd. (Nanjing, China). The sand used in this experiment was ISO standard sand produced by Xiamen ISO Standard Sand Co., Ltd. (Xiamen, China), and the water used was tap water.

### 2.2. Experimental Methods

(1) Water requirements of normal consistency, setting time, and compressive strength.

The water requirements of normal consistency and setting time were tested in accordance with GB/T1346-2011 [14]. The raw materials were weighed according to the mix proportion in Table 2, and the cement paste was stirred evenly by an NJ-160A cement paste mixer, quickly put into a 20 mm × 20 mm × 20 mm cubic mold, tamped and vibrated on a vibration table, taken down and scraped, and marked. Finally, the mold was put into a constant-temperature (20 ± 2 °C) and -humidity (>95%) curing chamber for curing for 30 min, 1 h, and 4 h, respectively. Subsequently, the sample was taken out and demolded, and its compressive strength was determined via a CMT5505 microcomputer control electron universal testing machine.

(2) Instrumental analyses.

The phase analysis of hydration products was performed by the D8-ADVANCE X-ray diffractometer of German Brooke Company (Saarbrücken, Germany). The hydration rate and hydration heat were tested by a TAM Air eight-channel isothermal microcalorimeter produced by Retrac HB Company (Stockholm, Sweden). The SEM was tested by a German ZEISS (Oberkochen, Germany) EVOLS15 scanning electron microscope.

## 3. Results and Discussion

### 3.1. Effects of Li_2_CO_3_ and Superplasticizer on Setting Time and Ultra-Early Strength of AYBFC

**Figure 2:**
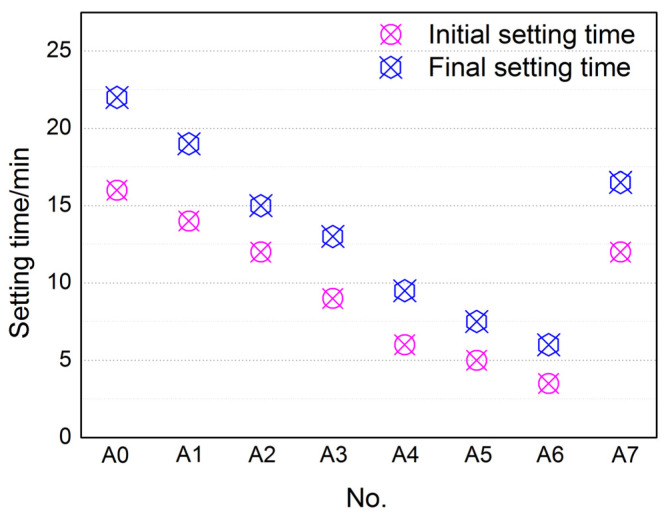
Effects of Li_2_CO_3_ and superplasticizer on setting time of AYBFC.

It can be seen from Figure 2 that both the initial and final setting time of AYBFC were accelerated with the increase of Li_2_CO_3_ content within a certain range of dosage. The initial and final setting time reduced from 16 and 22 min to about 4 and 6 min, respectively, when the dosage of Li_2_CO_3_ increased from 0.00 wt.% to 0.06 wt.%. The results indicated that the setting time of AYBFC could be accelerated by adding Li_2_CO_3_. When the Li_2_CO_3_ content was 0.05 wt.%, the initial and final setting time were about 5 and 8 min. After adding 0.35 wt.% polycarboxylate superplasticizer, the initial and final setting time increased to about 12 and 18 min, indicating that the polycarboxylate superplasticizer had the effect of delaying the setting time.

**Figure 3:**
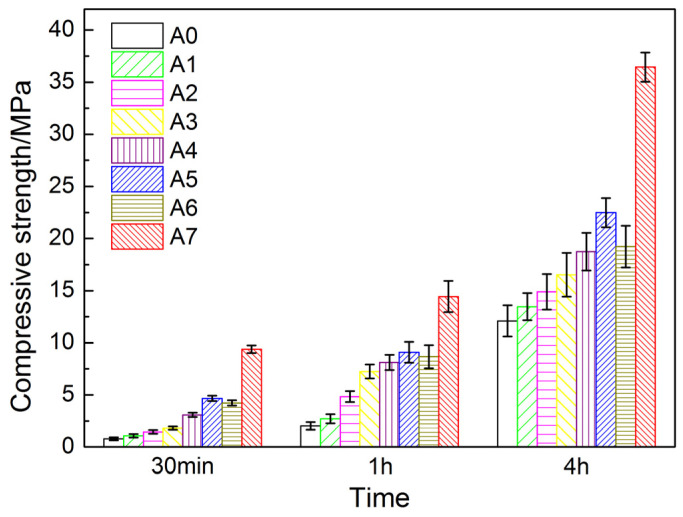
Effects of Li_2_CO_3_ and superplasticizer on ultra-early strength of AYBFC.

It can be seen from Figure 3 that the ultra-early compressive strength of AYBFC increased at first and then decreased with the increase in the content of Li_2_CO_3_. When the content of Li_2_CO_3_ was 0.05 wt.%, the compressive strength of AYBFC reached the highest value, and its compressive strength at 30 min, 1 h, and 4 h reached 4.67, 9.10, and 22.49 MPa, respectively, which were 498.7%, 348.3%, and 85.9% higher than that of the blank sample. This meant that the ultra-early compressive strength of AYBFC was significantly improved by an appropriate amount of Li_2_CO_3_, which was because of the very strong accelerating effect of Li on the start of the reaction [15]. This has already been reported in several studies for CSA [16] and for calcium aluminate cement [17,18,19,20].

On the above basis, 0.35 wt.% superplasticizer was co-doped with 0.05 wt.% Li_2_CO_3_, which markedly improved the strength of AYBFC at different ages. Specifically, the compressive strength at 30 min, 1 h, and 4 h were 101.1%, 58.7%, and 62.1% higher than that of the sample only doped with 0.05 wt.% Li_2_CO_3_. The doping of the superplasticizer inhibited the hydration of AYBFC, but it facilitated the electrostatic repulsion of the cement particles, and the water molecules wrapped by the cement particles were released, improving the fluidity of the slurry, reducing the water consumption, compacting the matrix [21], and further enhancing the compressive strength.

### 3.2. Effects of Li_2_CO_3_ and Superplasticizer on Micro-Composition and Structure of AYBFC

#### 3.2.1. Hydration Analysis

**Figure 4:**
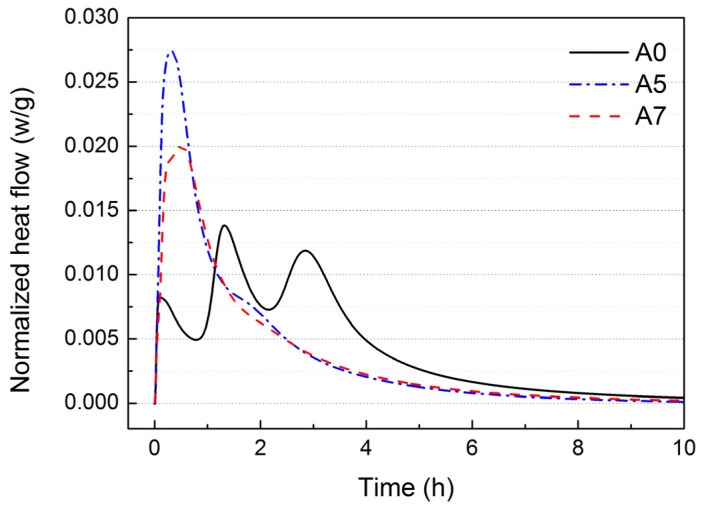
Hydration rate of each sample.

**Figure 5:**
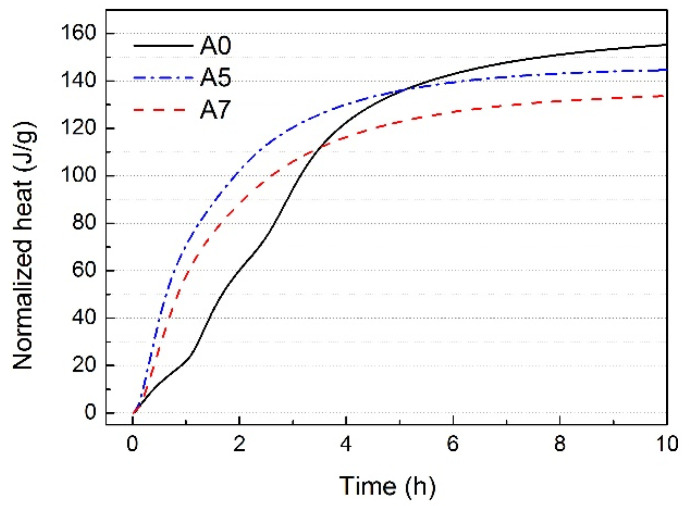
Hydration heat of each sample.

AYBFC has four exothermic peaks [12]. Except the first exothermic peak caused due to heat released by dissolution when C_4_A_3_$, C_3_S, and dihydrate gypsum came in contact with water, which was not easily observed because of the overlap, the other exothermic three peaks of the blank sample can clearly be seen in Figure 4. The second and third exothermic peaks were caused by the hydration reaction of ye’elimite (anhydrous barium calcium sulphoaluminate and C_4_A_3_$, collectively referred to as C_4_A_3_$ in this study). The fourth exothermic peak was a comprehensive exothermic peak mainly related to C_3_S. Compared with the blank sample, the four exothermic peaks of A5 and A7 all appeared in advance due to the presence of Li_2_CO_3_, and were nearly combined into one exothermic peak. It can be seen from Figure 5 that the hydration rate and early hydration heat release of AYBFC were the fastest due to 0.05 wt.% Li_2_CO_3_, but, by contrast, if 0.35 wt.% superplasticizer was doped, the hydration rate and hydration heat release of AYBFC were slowed down. This was consistent with the experimental results of the setting time, which was associated with the inhibitory effect of superplasticizer on the cement hydration reaction and the action mechanism for the generation of hydration products [22,23].

#### 3.2.2. XRD and SEM Analysis

**Figure 6:**
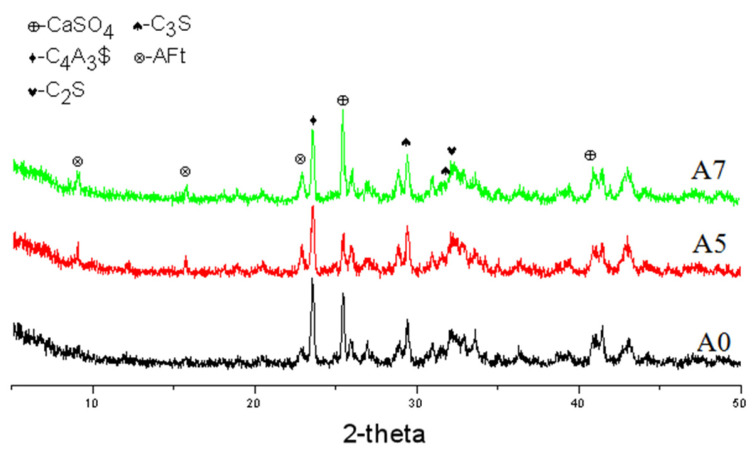
XRD patterns of each sample after hydration for 30 min.

**Figure 7:**
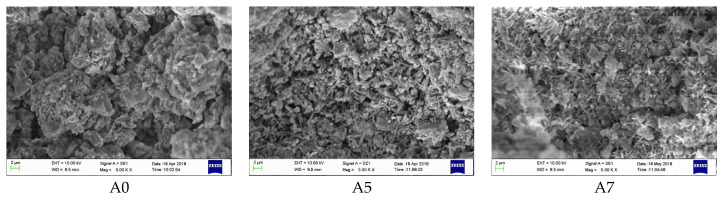
SEM images of each sample after hydration for 30 min.

It can be seen from Figure 6 that the diffraction peaks of clinker mineral phases C_4_A_3_$, C_3_S, C_2_S, and cement admixture gypsum were very clear in the A0 spectrum of the blank sample at 30 min of hydration, but without apparent AFt (ettringite; its chemical formula is 3CaO·Al_2_O_3_·3CaSO_4_·32H_2_O) diffraction peaks of hydration products, indicating a quite low hydration degree. By contrast, clear AFt diffraction peaks of samples A5 and A7 were observed, showing that the hydration of AYBFC was promoted by both the single doping of Li_2_CO_3_ and co-doping of Li_2_CO_3_ and superplasticizer. Comparing the images in Figure 7, it can be seen that almost no needle- or rod-like AFt was generated in the blank sample A0 due to the low hydration degree, and the hardening body was discontinuous with many pores. A large number of AFts were produced in samples A5 and A7; in particular, those in A5 were needle- or rod-shaped, being both large and thick, which was helpful to improve the ultra-early strength, but disadvantageous to the later strength [16]. The AFt in A7 was needle-shaped with a relatively small size. In addition, the matrix compactness of the samples could be clearly observed and sorted as A7 > A5 > A0, which was consistent with the results of compressive strength.

**Figure 8:**
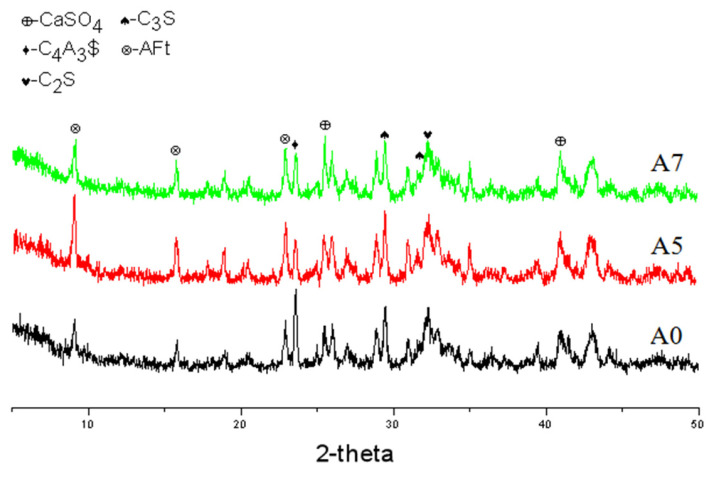
XRD patterns of each sample after hydration for 4 h.

**Figure 9:**
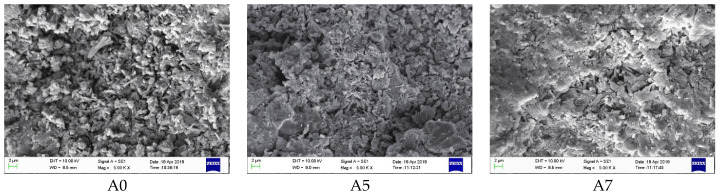
SEM images of each sample after hydration for 4 h.

As shown in Figure 8 and Figure 9, after hydration for 4 h, the diffraction peaks of the clinker mineral phase and gypsum in each sample decreased, while the AFt diffraction peak increased clearly, indicating that the hydration degree of each sample increased clearly; in particular, the AFt diffraction peak of sample A5 was the sharpest, followed by sample A7, which was also consistent with the results of the hydration rate. At this time, many AFts had been formed in the blank sample, but the matrix was still loose. Many gel products had been produced in samples A5 and A7, which wrapped the AFt to form a continuous structure, and the matrix was relatively compact. Evidently, the matrix compactness of A7 was the highest.

## 4. Conclusions

(1) An appropriate amount of Li_2_CO_3_ can significantly improve the ultra-early compressive strength of AYBFC. At the dose of 0.05 wt.%, the compressive strength of AYBFC reached the highest value, and its compressive strength at 30 min, 1 h, and 4 h increased by 498.7%, 348.3%, and 85.9%, respectively, compared with that of the blank sample.

(2) If 0.35 wt.% superplasticizer was co-doped with 0.05 wt.% Li_2_CO_3_, the strength of AYBFC at each age was markedly enhanced, and its compressive strength at 30 min, 1 h, and 4 h was 101.1%, 58.7%, and 62.1% higher than that of the sample only doped with 0.05 wt.% Li_2_CO_3_.

(3) The ultra-early compressive strength of AYBFC was improved most clearly by co-doping Li_2_CO_3_ with superplasticizer. First, an appropriate amount of Li_2_CO_3_ could promote the hydration of AYBFC and the early generation of hydration products. Second, the superplasticizer could inhibit the hydration reaction of the cement and the generation of hydration products, and it could strengthen the matrix compactness, which was beneficial for the development of strength.

## Figures and Tables

**Table 1:** Mineral composition of AYBFC clinker (wt.%).

Clinker Mineral	C_4_A_3_$	C_3_S	C_2_S	Ferrite
Content proportion	48.5	11.7	32.6	7.2

**Table 2:** Mix proportion of each specimen.

No.	Li_2_CO_3_/wt.%	Polycarboxylate Superplasticizer/wt.%	W/C
A0	0.00	/	0.31
A1	0.01	/	0.31
A2	0.02	/	0.31
A3	0.03	/	0.31
A4	0.04	/	0.31
A5	0.05	/	0.31
A6	0.06	/	0.31
A7	0.05	0.35	0.27

## Data Availability

Data are contained within the article.

## References

[1] Tan H., Zhang X., He X., Guo Y., Deng X., Su Y., Yang J., Wang Y. (2018). Utilization of lithium slag by wet-grinding process to improve the early strength of sulphoaluminate cement paste. J. Clean. Prod..

[2] Wang Y.M., Su M.Z., Zhang L. (1999). Sulphoaluminate Cement.

[3] Zhang Y., Wang Y., Li T., Xiong Z., Sun Y. (2018). Effects of lithium carbonate on performances of sulphoaluminate cement-based dual liquid high water material and its mechanisms. Constr. Build. Mater..

[4] Tan H., Li M., He X., Su Y., Yang J., Zhao H. (2021). Effect of wet grinded lithium slag on compressive strength and hydration of sulphoaluminate cement system. Constr. Build. Mater..

[5] Han J.G., Yan P.Y. (2010). Influnce of lithium compound on sulphoaluminate cement hydration process. J. Chin. Ceram. Soc..

[6] Paaver P., Järvik O., Kirsimä K. (2021). Design of high volume CFBC fly ash based calcium sulphoaluminate type binder in mixtures with ordinary Portland cement. Materials.

[7] Yoon H.N., Seo J., Kim S., Lee H.K., Park S. (2021). Hydration of calcium sulfoaluminate cement blended with blast-furnace slag. Constr. Build. Mater..

[8] Gao D., Meng Y., Yang L., Tang J., Lv M. (2019). Effect of ground granulated blast furnace slag on the properties of calcium sulfoaluminate cement. Constr. Build. Mater..

[9] Yang W., Rao M.J., Deng Q.S., Wang F. (2024). Effects of CuO doping on the calcination and hydration performance of alite-belite-ferrite-ye’elimite cement. Dev. Built Environ..

[10] Du P., Li X., Zhou Z., Lu X., Zhang X., Xu D., Cheng X. (2021). Preparation and properties of alite modified calcium sulfoaluminate cement. Adv. Cem. Res..

[11] Lu X., Ye Z., Wang S., Du P., Li C., Cheng X. (2018). Study on the preparation and properties of belite-ye’elimite-alite cement. Constr. Build. Mater..

[12] Li P., Ma Z., Zhang Z., Li X., Lu X., Hou P., Du P. (2019). Effect of gypsum on hydration and hardening properties of alite modified calcium sulfoaluminate cement. Materials.

[13] Lu X.L. (2019). Study on Mineral Composition, Structure and Properties of Alite Modified Calcium Sulphoaluminate Cement Clinker.

[14] (2011). Test Methods for Water Requirement of Normal Consistency, Setting Time and Soundness of the Portland Cements.

[15] Manninger T., Jansen D., Neubauer J., Goetz-Neunhoeffer F. (2019). Accelerating effect of Li_2_CO_3_ on formation of monocarbonate and Alhydroxide in a CA-cement and calcite mix during early hydration. Cem. Concr. Res..

[16] Cau Dit Coumes C., Dhoury M., Champenois J.B., Mercier C., Damidot D. (2017). Physicochemical mechanisms involved in the acceleration of the hydration of calciumsulfoaluminate cement by lithium ions. Cem. Concr. Res..

[17] Matusinović T., Čurlin D. (1993). Lithium salts as set accelerators for high alumina cement. Cem. Concr. Res..

[18] Rodger S.A., Double D.D. (1984). The chemistry of hydration of high alumina cement in thepresence of accelerating and retarding admixtures. Cem. Concr. Res..

[19] Damidot D., Rettel A., Capmas A. (1996). Action of admixtures on Fondu cement: Part 1. Lithium and sodium salts compared. Adv. Cem. Res..

[20] Damidot D., Rettel A., Sorrentino D., Campas A. (1997). Action of admixtures on fondu cement: II. Effect of lithium salts on the anomalous setting time observed for temperatures ranging from 18 to 35 °C. Adv. Cem. Res..

[21] El-Didamony H., El-Sokkari T.M., Khalil K.A., Ahmed I. (2012). Hydration mechanisms of calcium sulphoaluminate C_4_A_3_$ over-bar, C_4_A$ over-bar phase and active belite β-C_2_S. Ceram. Silikáty.

[22] Júnior L.U.D.T., Lima G.T.D.S., Silvestro L., Ruviaro A.S., Gleize P.J.P., de Azevedo A.R.G. (2023). Influence of polycarboxylate superplasticizer and calcium sulfoaluminate cement on the rheology, hydration kinetics, and porosity of Portland cement pastes. J. Build. Eng..

[23] Barneoud-Chapelier A., Le Saout G., Azéma N., El Bitouri Y. (2022). Effect of polycarboxylate superplasticizer on hydration and properties of belite ye’elimite ferrite cement paste. Constr. Build. Mater..

